# Vulnerability of cotton subjected to hail damage

**DOI:** 10.1371/journal.pone.0210787

**Published:** 2019-01-30

**Authors:** Yaojie Yue, Lan Zhou, A-xing Zhu, Xinyue Ye

**Affiliations:** 1 Key Laboratory of Environmental Change and Natural Disaster of Ministry of Education; School of Geography, Faculty of Geographical Science, Beijing Normal University, Beijing, China; 2 Jiangxia Jinkou High School, Wuhan, China; 3 Department of Geography, University of Wisconsin-Madison, Madison, Wisconsin, United States of America; 4 Jiangsu Center for Collaborative Innovation in Geographical Information Resource Development and Application and School of Geography, Nanjing Normal University, Nanjing, China; 5 Urban Informatics & Spatial Computing Lab, Department of Informatics, New Jersey Institute of Technology, Newark, United States of America; Fred Hutchinson Cancer Research Center, UNITED STATES

## Abstract

This paper establishes the quantitative relationships between hail fall parameters and crop damages by examining the impacts of 49 hail hazard scenarios on cotton in the bud stage and boll stage. This study utilizes simulated cotton hail hazard to analyze the following data: hail size, hail fall density, and crop damages (i.e., defoliation rate, branch breaking rate, and the fruit falling rate). The results are as follows: 1) cotton vulnerability increased via an increase in crop damages as the hail hazard magnitude increased; 2) crop damages exhibit significant logistic relationships with hail diameter and hail fall density, and the fit was better at the bud stage than at the boll stage; 3) cotton is more vulnerable to hail hazard at the bud stage than at the boll stage, and the bud stage is a critical period for cotton hail disaster prevention and mitigation; and 4) damages to cotton plant at the bud stage and boll stage were less sensitive to hail size from hail fall density. Thus, we suggest that hail diameter can be used as the priority indicator to predict hail-induced crop damages. These results provide a sound basis for developing a comprehensive evaluation of hail damage in cotton, which is crucial for promoting sustainable cotton production. We recommend that the impacts of hail-induced crop damages on yield and fiber quality need to be addressed further in future studies.

## Introduction

It has been reported that the total number of severe weather events, including hail, decreased significantly throughout most of China over the past and recent decades [1–4]. Fewer hail days are expected over most areas of the US in the future [5]. However, damage from hailstorms may increase in frequency and severity with global warming [6, 7], which may cause a shift toward the more frequent occurrence of larger hail [5]. Crops are highly vulnerable to hail strike, and hail can induce severe agricultural losses [1, 8–14]. Therefore, vulnerability analyses of crops subjected to hail falls is an essential step for reducing the consequences of hail events [15]. Analyzing the impact of hail falls on vulnerable crops is a crucial scientific issue for promoting sustainable agriculture, given the trend of increasing hailstorms. However, there are few well-established procedures for such an analysis in China and around the world.

‘Vulnerability’ refers to sensitivity to damage from hazards and is regarded as the standard for evaluating loss or damage degrees of a hazard-affected body [16, 17]. Scientists often view vulnerability in terms of the likelihood of specific scenarios and the associated impacts on the built environment (e.g., [15, 18, 19]). For instance, Shi (2011) [20] argues that the vulnerability of a hazard-affected body can be represented by a function between hazard intensity and corresponding damages/losses. However, one of the challenges of vulnerability research is the determination of the relationship between hazard intensity and corresponding damages or losses.

To quantify this relationship, experimental methods, including leaf cutoff and field experiments for simulating hail fall and damages, have commonly been adopted. However, these methods are still in the early adoption phase. In the leaf cutoff method, vulnerability is quantified by the relationship between the defoliation rate and crop yield losses, and the defoliation rates are obtained by cutting off leaves to simulate the effect of hail falls (e.g., [21–23]). However, the instruments commonly used in these studies are grass trimmers [21, 22], hand sickles [21, 24], or high pressure washers [23], and these instruments cause crop damages that are quite different from those caused by natural hail fall. For example, hail fall characters, such as diameter, amount, and duration, could hardly be observed. Furthermore, the effects of strong winds and rain, which usually accompany hail, are excluded from these experiments. Therefore, it is essential to design reliable experimental equipment and methods to reproduce natural hail falls and reveal crop vulnerability to hail falls.

The process of natural hail fall can be simulated to observe relevant crop damages and to analyze the effects of different damage intensities on crop yield and quality, e.g., cotton [25–29], potato [23, 30, 31], Onion [21], soybean [24, 32], wheat [32, 33], lentil [34], and guar [22]. For cotton, Lane (1959) [25] simulated the effect of hail strike on cotton yield and reported no relationship between the defoliation rate and the ultimate cotton yield. Li (1993) [27] as well as Li and Zhang (1993) [35] studied the relationship between cotton yield and crop damage through experiments and field surveys. These studies have proved that the effects of hail fall-induced damages to crop yield could be evaluated quantitatively using hail simulation methods. However, the experimental equipment and materials at this stage are too simple to simulate hail fall parameters such as hail diameter, hail fall density, and landing speed [36]. For example, Lane (1959) [25] used equipment that shot crushed ice at plants to simulate hail strikes on cotton, but the working parameters of the equipment are unclear. Seino (1985) [32] produced an apparatus that reproduced hail fall by freely dropping stones from a 3.4 m height. Li (1993) [27] as well as Li and Zhang (1993) [35] manually struck cotton with a stick and threw stones. These shortcomings in hail fall simulation equipment and methods make it very difficult to accurately measure the diameter, landing speed, or number of stones per unit area. These are key parameters for revealing the hail hazard intensity.

China is one of the largest cotton-producing countries in the world [37] and is subjected to severe hail disasters [12, 38]. The goal of this study was to propose an experimental approach that helps quantify the impact of hail on cotton, and then reveal the effects of hail parameters on different types of crop damage. In the following sections, we first describe the methodological framework. Then, the proposed method will be applied in a case study to analyze the effects of hail parameters on cotton damage. Finally, we discuss how the research findings can be used broadly.

## Methodology

In this paper, the vulnerability of cotton to hail is defined as the quantitative relationship of damage to cotton leaves, branches, buds, and bolls by corresponding hail hazard intensity, that is, the focus is on assessing the damage to cotton morphology. The impact of hail-induced damages on cotton yields was also observed in our study, and these results will be presented in a future paper.

### Cotton hail hazard experimental approach

#### Experimental field and cotton variety

The field study was carried out on private farmland, and the owner of the land provided consent for the study to be conducted on this site. The field studies did not involve endangered or protected species. The experimental field, covering 0.154 ha, is located in Rongjiazhuang Village, Sanhe County, Hebei Province. The study area is in a traditionally cotton planting region within the North China plain, approximately 60 km northeast of Beijing (please refer to Fig 1 in [39] for the location and land use map of Sanhe County). This area is characterized as temperate monsoon with a hot and rainy summer as well as a cold and dry winter [40], and the area has frequent hail events impacting production [1].

The variety of cotton used for the experiment was GK45, a commonly used variety in this region. Cotton was planted at a density of 45,000 plants/ha with row spacing of 80 cm and plant spacing of 30 cm. The entire experimental field was treated with a uniform planting and management system to ensure consistent growth and development in the experimental cotton.

Using the proposed experimental apparatus at the set height and output angle, the area covered by the simulated hail hazard is approximately 1 m^2^. Considering the cotton planting specifications (row spacing of 80 cm and plant spacing of 30 cm), we set the experimental plot size as 1.0 m×1.0 m. Experimental plots with an area of 1.0 m^2^ were created using the split-plot method. Therefore, there are approximately four to five plants per plot.

#### Hail hazard scenarios

Hail size, hail fall density (amount of hailstones per area), and landing speed of hailstones are the indicators that are typically used to describe hail hazard intensity. According to Lei et al. (1978) [41], Guo and Yan (1999) [42], and Duan (2009) [13], the hail fall density ranges mostly from 100 to 500 ice balls per m^2^, with a hail diameter of 0.5–5.0 cm. Therefore, 7 hail fall densities (100, 150, 200, 300, 400, 450, and 500 ice balls/m^2^) and 7 hail ball diameters (1.0 cm, 1.5 cm, 2.0 cm, 2.5 cm, 3.0 cm 4.0 cm, and 5.0 cm) were chosen to simulate 49 hail hazard scenarios in total.

The field experiment was conducted at the bud stage (i.e., the period from the bud to first flowering) and the boll stage (i.e., the period from the first flowering to boll opening). In the study area, the bud stage usually lasts from early June to early July (approximately 4 weeks), and the boll stage generally runs from early July to late August (approximately 8 weeks) [43]. Considering the long duration of the bud stage and boll stage, the tolerance of cotton to hail attack also changed during this period. Therefore, the experiment was repeated 2 and 4 times in the bud stage and boll stage, respectively (once every two weeks). These repeated measures were performed to comprehensively reflect the vulnerability of cotton to hail strike in different growth stages. In the experiment, each hail hazard scenario is replicated 3 times, and 3 control plots were created. Therefore, there were a total of 900 experimental plots.

The landing velocity of hail balls varies with diameter (Table 1). Statistically, hail diameter mostly ranges from 3.0 cm to 7.0 cm. This range accounts for 60.7% of hail disaster records in China. Therefore, it is reasonable to set the hail ball landing terminal velocity at over 25 m/s but less than 45 m/s in most cases. The International Electrotechnical Commission (IEC) sets the hail ball landing speed at approximately 25.0 m/s in the anti-hail tests of Photovoltaic (PV) modules [44]. Therefore, we set the hail ball landing terminal velocity at 25 m/s in this experiment.

**Table 1:** Relationship of hail ball diameter and terminal velocity.

Hail ball diameter (cm)	0.6	1.0	2.0	3.0	4.0	6.0	10.0	14.0	20.0	30.0
Terminal velocity (m/s)	11	14	20	25	28	35	45	53	63	77

Data source: [41]

The hail ball, that is, ice ball, was created in a freezer using fresh water and an ice-ball mold. The average density of the ice balls was 0.975 g/cm^3^. Ice balls of different diameters were prepared ahead of the experiment. During the experiment, the hail balls were transferred from the freezer into insulated boxes and were then transported to the field.

#### Experimental apparatus and its working parameters

An experimental apparatus to simulate hail fall was designed [45, 46]. Its operating principle is as follows: an internal combustion engine is used to drive a centrifugal fan to produce a high-pressure blast of air, the air accelerates and transmits hail balls along a transmission pipe, and the ice balls are then propelled at the hail-affected body at a certain speed.

In this case, the maximum power and nominal speed of the internal combustion engine is 16.2 kW and 2,200 rpm, respectively. The centrifugal fan has a flow of 1,174 to 2,062 m^3^/h, a total pressure of 4,603 to 4,447 Pa, and a nominal speed of 2,900 rpm. The height of the apparatus platform is 125.0 cm above ground, and the height of the hailstorm outlet is set to approximately 155.0 cm. The blast transmission pipe is 2.5 m long, with a diameter of 15 cm. The apparatus can propel hail balls with diameters between 0.5 and 10.0 cm. Its shooting direction could be vertically adjusted between 0° and 120°, and it could be adjusted horizontally between 0° and 180°. This setup ensures that the hail balls are shot at the specific cotton plants within the specified range. The outlet air velocity of the blast transmission pipe is 22.0 m/s when the engine is idled at 800 rpm, and the air velocity is 48.0 m/s in the case of a maximum speed of 2,200 rpm. Therefore, the hail ball speed could range from 20.0 m/s to 45.0 m/s. If the engine speed is controlled between 1,050 rpm and 1,150 rpm using a digital tachometer (with a testing range between 5 and 99,999 rpm at a resolution of 0.1 rpm), the hail speed could be stably controlled at approximately 25.0 m/s. This speed matches the level of hail terminal velocity.

#### Experimental procedures

Following Yue et al. (2013; 2015) [46, 47], the experimental processes included five steps: 1) experimental plots were selected, marked, and numbered; 2) the apparatus and ice balls were prepared; 3) the quantity of leaves, branches, and fruits (buds and bolls) for each cotton plant were recorded individually in each plot; 4) simulated hail fall was conducted through the apparatus according to hail fall scenarios; and 5) hail fall-induced cotton damage in each plot was recorded, and the number of leaves, branches, buds, and bolls of each cotton plant in each plot was individually quantified.

### Cotton vulnerability analyses

In the present study, cotton vulnerability analysis aimed to reveal the effects of hail fall parameters on various indicators of cotton damage, such as a defoliation rate (DR), branch breaking rate (BBR), and fruit (including bud and boll) falling rate (FFR). The analyses use hail of different sizes and different densities at the bud stage and boll stage. Based on the experimental data, the damage indices of DR, BBR, and FFR are calculated using the following equations:

$$DR=\frac{Prel-Poel}{Prel}\times 100\%,$$ (1)

where DR is the defoliation rate, Prel is the pre-experiment number of leaves, and Poel is the post-experiment number of leaves.

$$BBR=\frac{Preb-Poeb}{Preb}\times 100\%,$$ (2)

where BRR is the branch breaking rate, Preb is the pre-experiment number of branches, and Poeb is the post-experiment number of branches.

$$FFR=\frac{Pref-Poef}{Pref}\times 100\%,$$ (3)

where FFR is the fruit falling rate, Pref is the pre-experiment number of fruits, and Poef is the post-experiment number of fruits. In the equations above, the numbers of cotton leaves, branches, and fruits before and after the experiment are the statistical average at the scale of 1.0 m×1.0 m experimental plots for each hail hazard scenario in every growth stage.

The use of a logistic function is widely accepted for expressing relationships of hail hazard and its damages on crops [33, 48]. This paper adopts this practice and establishes logistic functions of hail fall parameters with cotton damages (including DR, BBR, and FFR). The least squares method was utilized to calculate the regression coefficients, and the adjusted coefficient of determination (R^2^adj) was used to test the effectiveness of the logistic function with a 0.05 confidence level. We also used the residual error sum of squares (RSS) to compare the predicted crop damage values with the observed values to test the accuracy of the logistic functions. Smaller RSS values indicate a superior logistic function fit, given the same dataset.

Additionally, the average variation coefficients of cotton damages with hail sizes and hail fall densities at the bud stage and boll stage were calculated to analyze the degree of vulnerability for the leaves, branches, and fruits subjected to various hail sizes and densities.

## Results

### Cotton damages and hail size

#### Effect of hail size on the defoliation rate

The results show that the defoliation rate rises with increased hail size at the bud stage and boll stage, regardless of the hail fall density (S1 Fig). In most cases, the defoliation rate increased with the increase in hail fall density given the same hail size. The minima defoliation rates were 0.19 at the bud stage and 0.13 at the boll stage, whereas the maxima rates were 0.88 at the bud stage and 0.68 at the boll stage. Both the minimum and maximum defoliation rates at the bud stage were greater than those at the boll stage, which suggests that the cotton leaves are more vulnerable to hail size at the bud stage than at the boll stage.

**Fig 1:**
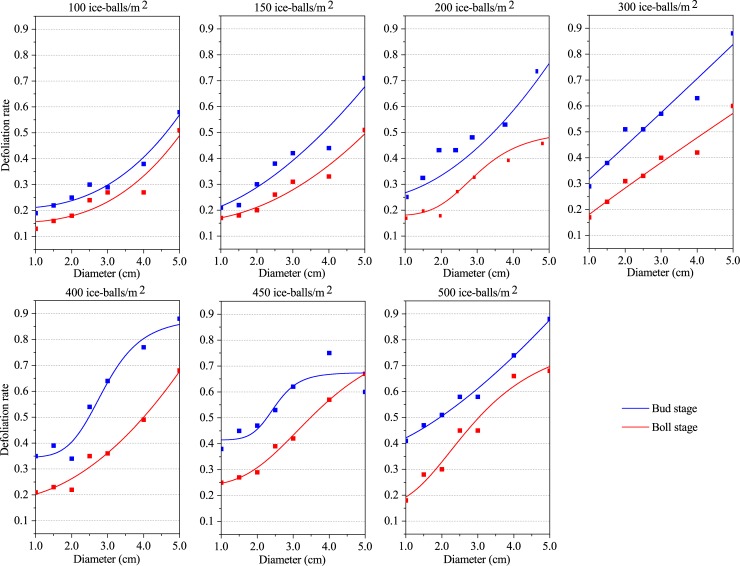
Logistic relationship between the defoliation rate and hail size.

A significant logistic functional relationship was determined to occur between the defoliation rate and the hail diameter (Fig 1, Table 2). The R^2^adj of the fitted logistic function between the hail size and the defoliation rate remained above 0.85, except for two cases. This suggests a significant logistic functional relationship lies between the defoliation rate and the hail diameter, both at the bud stage and boll stage. Meanwhile, the RSS was less than 0.03 at the bud stage, whereas it was less than 0.008 at the boll stage (S1 Table). This implies that the logistic functional relationship between the defoliation rate and the hail diameter was much stronger in the boll stage than at the bud stage.

**Table 2:** Regression parameters of the logistic functions between the defoliation rate and hail size.

Regression parameter		100 particles/m^2^	150 particles/m^2^	200 particles/m^2^	300 particles/m^2^	400 particles/m^2^	450 particles/m^2^	500 particles/m^2^
A_1_	Bud stage	0.21	0.18	0.24	0.20	0.35	0.41	0.37
	Boll stage	0.15	0.16	0.18	0.07	0.18	0.24	0.16
A_2_	Bud stage	2528.67	3637.68	4270.49	109587.14	0.88	0.67	2686.34
	Boll stage	4406.47	1576.91	0.51	8567.55	320.11	0.85	0.83
X_0_	Bud stage	131.96	1009.62	549.06	583961.54	2.92	2.50	1756.71
	Boll stage	149.09	342.61	3.03	175557.52	129.72	3.81	3.00
p	Bud stage	2.70	1.68	1.92	1.03	5.51	7.52	1.46
	Boll stage	2.79	2.00	4.61	0.93	1.99	3.21	2.70
R^2^_adj_	Bud stage	0.95	0.87	0.74	0.88	0.94	0.68	0.98
	Boll stage	0.83	0.92	0.95	0.91	0.96	0.99	0.93

Note: logistic function: y = A_2_ + (A_1_-A_2_)/(1 + (x/x_0_)^p).

#### Effect of hail size on the branch breaking rate

The results show that branch damages at the bud stage and boll stage also rise with increasing hail size (S2 Fig). In most cases, the branch breaking rate and defoliation rate increase with the increase in hail fall density given the same hail size. The branch breaking rate varies significantly at the bud stage and boll stage. The minimum branch breaking rate is 0.15 at the bud stage and 0.07 at the boll stage. However, the maximum branch breaking rate is 0.99 at the bud stage and only 0.62 at the boll stage. When the average hail density was 300 particles/m^2^, the variation in the branch breaking rate ranged from 0.22 to 0.75 during the bud stage and from 0.09 to 0.48 during the boll stage. The variation in the minimum and maximum branch breaking rates was the same as that of the defoliation rate, indicating that cotton branches are more vulnerable to large hail stones during the bud stage than during the boll stage.

**Fig 2:**
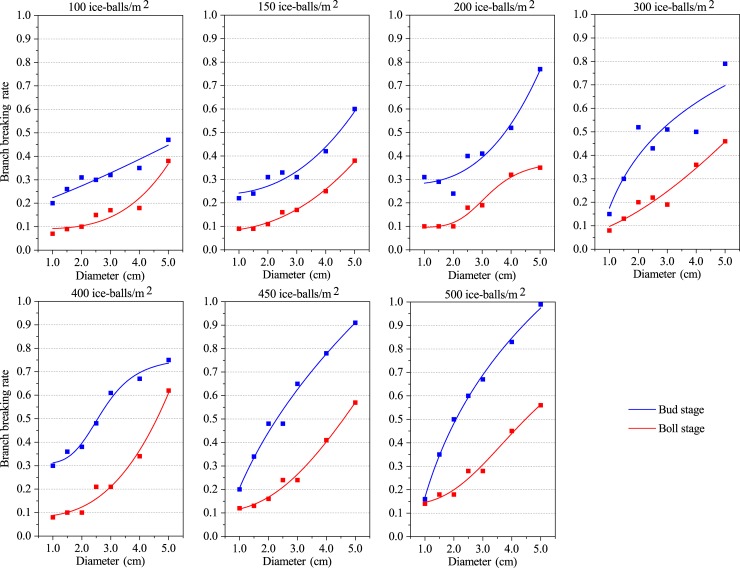
Logistic relation between the branch breaking rate and hail size.

The quantitative relationship between hail size and branch breaking rate is shown in Fig 2 and Table 3. A significant logistic function was found between the branch breaking rate and the hail diameter at both the bud stage and boll stage. The R^2^adj at both the bud stage and boll stage exceeded 0.65, and it rose above 0.87 at the boll stage. Meanwhile, the results indicate that the RSS is less than 0.05 at both the bud stage and boll stage, and it is always less than 0.006 at the boll stage (S1 Table). This suggests that the simulation results based on the logistic relationship between cotton branch vulnerability and hail size at the boll stage has relatively high accuracy.

**Table 3:** Regression parameters of logistic functions between the branch breaking rate and hail size.

Regression parameter		100 particles/m^2^	150 particles/m^2^	200 particles/m^2^	300 particles/m^2^	400 particles/m^2^	450 particles/m^2^	500 particles/m^2^
A_1_	Bud stage	0.18	0.24	0.28	-5.59	0.31	-0.35	-1.37
	Boll stage	0.09	0.08	0.10	0.06	0.08	0.11	0.14
A_2_	Bud stage	9811.29	3637.68	390.88	9.21	0.76	17.26	9.32
	Boll stage	3365.80	1576.91	0.38	674.21	2107.66	2.19	0.93
X_0_	Bud stage	41359.84	1009.62	53.00	140.82	2.73	604.77	265.41
	Boll stage	82.78	342.61	3.24	717.16	98.89	8.33	4.74
p	Bud stage	1.17	1.68	2.83	0.09	4.60	0.53	0.32
	Boll stage	3.35	2.00	5.29	1.50	2.78	2.44	2.83
R^2^_adj_	Bud stage	0.81	0.87	0.90	0.65	0.97	0.97	0.99
	Boll stage	0.87	0.92	0.96	0.90	0.96	0.98	0.97

Note: logistic function: y = A_2_ + (A_1_-A_2_)/(1 + (x/x_0_)^p).

#### Effect of hail size on the fruit falling rate

The results show that the fruit falling rate rises at both the bud stage and boll stage with increasing hail size. Cotton leaves and branches exhibit a similar pattern (S3 Fig). The minimum fruit falling rate was 0.20 at the bud stage and 0.09 at the boll stage, and the maximum fruit falling rate was 0.99 at the bud stage and 0.69 at the boll stage. Using the average hail fall density of 300 particles/m^2^ and the hail diameter change from 1.0 cm to 5.0 cm as an example, the fruit falling rate ranged from 0.28 to 0.75 in the bud stage and from 0.14 to 0.55 in the boll stage. The above results indicate that the cotton fruits are more vulnerable to rising hail size during the bud stage than in the boll stage. It also shows that the lignification of cotton plants from bud stage to boll stage increases the ability of cotton to withstand hail strike.

**Fig 3:**
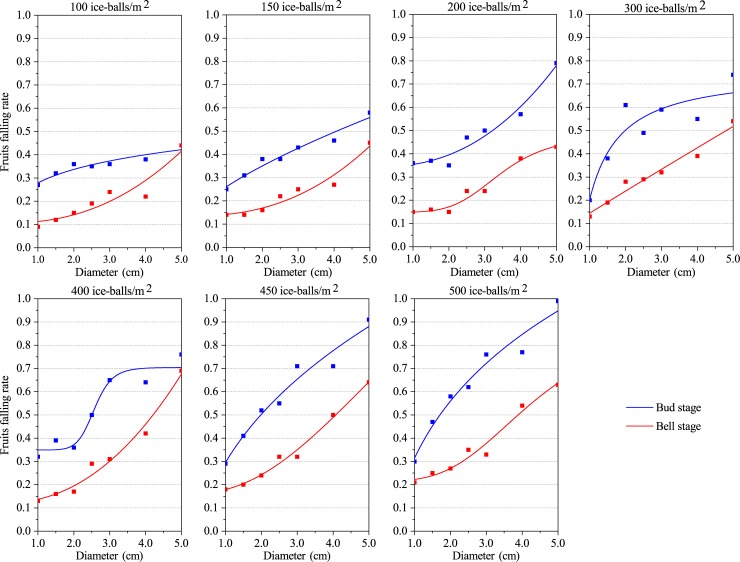
Logistic relationship between the boll falling rate and hail size.

A significant logistic relationship was found between the fruit falling rate and the hail diameter during both the bud stage and boll stage (Fig 3, Table 4). The R^2^adj at both the bud stage and boll stage exceeds 0.67. Furthermore, the R^2^adj exceeded 0.80 in the boll stage, which suggests that the fitting effect of the logistic function is better for the boll stage than for the bud stage. Meanwhile, the RSS was less than 0.04 at both the bud stage and boll stage; however, it is always less than 0.009 during the boll stage (S1 Table). This indicates that the logistic simulation of cotton fruit damages via hail size is more reliable at the boll stage.

**Table 4:** Regression parameters of the logistic equations between the boll falling rate and hail size.

Regression parameter		100 particles/m^2^	150 particles/m^2^	200 particles/m^2^	300 particles/m^2^	400 particles/m^2^	450 particles/m^2^	500 particles/m^2^
A_1_	Bud stage	-0.15	0.12	0.34	-93.26	0.35	-0.29	-0.81
	Boll stage	0.11	0.14	0.15	0.05	0.12	0.16	0.22
A_2_	Bud stage	2.09	143.60	1242.35	0.75	0.70	10.35	7.71
	Boll stage	3839.98	2176.94	0.48	38795.60	2134.34	1.85	0.91
X_0_	Bud stage	527.56	19129.49	150.85	0.01	2.58	434.31	284.77
	Boll stage	279.77	207.26	3.45	569429.94	210.55	7.51	4.36
p	Bud stage	0.23	0.70	2.33	1.15	10.51	0.47	0.33
	Boll stage	2.34	2.39	4.74	0.97	2.21	2.26	3.18
R^2^_adj_	Bud stage	0.80	0.92	0.93	0.67	0.89	0.92	0.93
	Boll stage	0.80	0.90	0.95	0.93	0.96	0.98	0.94

Note: logistic equation: y = A_2_ + (A_1_-A_2_)/(1 + (x/x_0_)^p).

From the relationship of hail diameter with the defoliation rate, the branch breaking rate, and the fruit falling rate, it can be concluded that the vulnerability of cotton plants increases with increasing hail size. This suggests that hail size might be an important factor for predicting cotton damages. Furthermore, it also indicates that cotton vulnerability to hail hazard decreases with the extension of the growth period, that is, cotton leaves and branches at the bud stage are more vulnerable to hail size than in the boll stage.

### Cotton damages and hail fall density

#### Effect of hail fall density on the defoliation rate

The results indicate that the defoliation rate increases with increasing hail fall density during the bud stage and boll stage (S4 Fig). Minima defoliation rates appear when the hail size is 1.0 cm: 0.19 during the bud stage and 0.13 during the boll stage. However, the maxima defoliation rates appear when the hail size is 5.0 cm: 0.88 during the bud stage and 0.68 during the boll stage. The average hail size was 2.71 cm. With the changing hail fall density, the defoliation rate ranges from 0.32 to 0.60 at the bud stage and from 0.25 to 0.44 at the boll stage. This suggests that cotton leaves are more vulnerable to hail fall density during the bud stage than during the boll stage, regardless of hail size.

**Fig 4:**
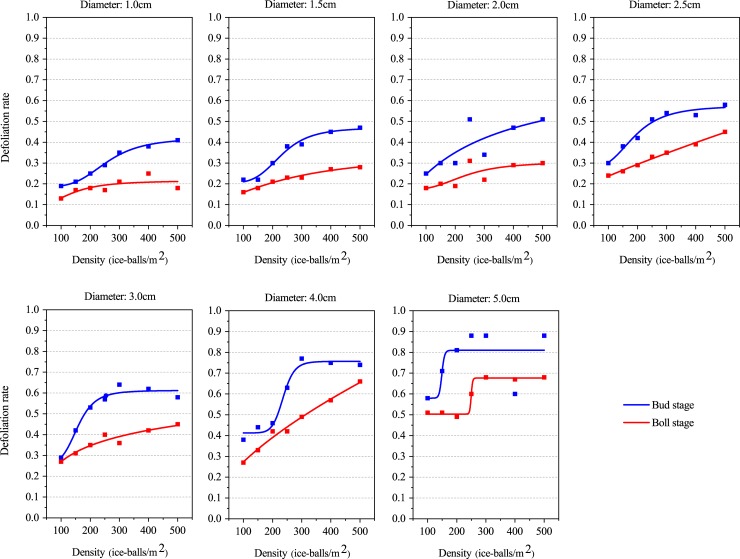
Logistic function between the defoliation rate and hail fall density.

A significant logistic relationship lies between the hail density and the defoliation rate at both the bud stage and the boll stage (Fig 4, Table 5). The R^2^adj is greater than 0.8 when the hail size was 1.5, 2.5, 3.0, and 4.0 cm, while it was less than 0.4 when the hail size was 1.0, 2.0, and 5.0 cm. This indicates that the logistic relation between the defoliation rate and hail density was not as good a fit as the relationship between the defoliation rate and hail size. Except for a very few cases, the RSS was less than 0.06 for both the bud stage and boll stage, and it was always less than 0.008 at the boll stage (S1 Table). This indicates that the simulation results based on the logistic relationship between cotton leaf damage and hail density during the boll stage exhibits relatively high accuracy.

**Table 5:** Regression parameters of logistic equations between the defoliation rate and hail fall density.

Regression parameter		1.0 cm	1.5 cm	2.0 cm	2.5 cm	3.0 cm	4.0 cm	5.0 cm
A_1_	Bud stage	0.19	0.20	0.08	0.27	0.26	0.41	0.58
	Boll stage	0.10	0.12	0.17	0.17	-1.75	0.07	0.50
A_2_	Bud stage	0.42	0.47	0.74	0.58	0.61	0.76	0.81
	Boll stage	0.22	0.35	0.30	9.03	1.40	40.27	0.68
X_0_	Bud stage	255.76	229.78	277.73	186.97	156.96	236.69	148.82
	Boll stage	139.25	278.45	229.86	22641.89	2.36	278482.15	249.43
p	Bud stage	4.11	4.61	1.04	3.52	5.30	12.45	33.05
	Boll stage	2.58	1.46	3.38	0.90	0.16	0.67	102.35
R^2^_adj_	Bud stage	0.99	0.96	0.35	0.94	0.94	0.96	-0.11
	Boll stage	0.17	0.97	0.25	0.99	0.84	0.97	0.99

Note: logistic equation: y = A_2_ + (A_1_-A_2_)/(1 + (x/x_0_)^p).

#### Effect of hail fall density on the branch injury rate

The branch injury rate increases with increased hail fall density at both the bud stage and the boll stage, except when hail diameter is 1.0 cm. When the hail size is greater than 4.0 cm, the branch injury rate increases even more quickly (S5 Fig). The minimum branch injury rate appears when the hail size is 1.0 cm: 0.15 during the bud stage and 0.07 during the boll stage. However, the maximum appears when the hail size is 5.0 cm: 0.99 during the bud stage and 0.62 during the boll stage. When the average hail size is approximately 2.71 cm and the hail density varies, the branch injury rate ranges from 0.32 to 0.58 during the bud stage and from 0.16 to 0.29 during the boll stage. Both the minimum and the maximum branch injury rates during the bud stage are greater than those during the boll stage, which indicates that the vulnerability of cotton branches to hail density during the bud stage is higher than that during the boll stage. This pattern was also found for the defoliation rate, which changes with the hail density.

**Fig 5:**
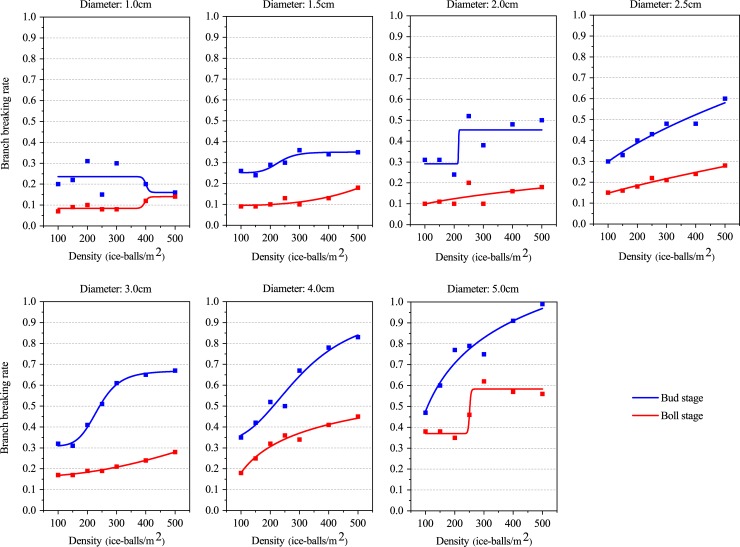
Logistic function between the branch injury rate and hail fall density.

A significant logistic relationship between hail fall density and branch injury rate at both the bud stage and the boll stage was obtained via curve fitting. The R^2^adj was below the 0.05 confidence level (Fig 5; Table 6). When the hail size is 1.5 cm, 2.5 cm, 3.0 cm, 4.0 cm, and 5.0 cm, the R^2^adj at both the bud stage and boll stage exceeded 0.7. Meanwhile, the RSS is less than 0.02 at both the bud stage and the boll stage (S1 Table). However, the R^2^adj was negative when the hail size is 1.0 cm during the bud stage and 2.0 cm during the boll stage, indicating that the logistic relationship between the hail fall density and the branch injury rate is poor for these two cases.

**Table 6:** Regression parameters of the logistic equations between the branch injury rate and hail fall density.

Regression parameter		1.0 cm	1.5 cm	2.0 cm	2.5 cm	3.0 cm	4.0 cm	5.0 cm
A_1_	Bud stage	0.24	0.25	0.29	0.13	0.31	0.33	-4.61
	Boll stage	0.08	0.09	0.04	0.10	0.16	-2.21	0.37
A_2_	Bud stage	0.16	0.35	0.45	33.58	0.67	0.97	1.99
	Boll stage	0.14	788.14	6.43	18.13	1.55	0.85	0.58
X_0_	Bud stage	400.58	229.04	215.17	545898.29	237.19	304.52	1.80
	Boll stage	396.38	9641.79	549205.67	161862.13	1660.62	3.29	250.76
P	Bud stage	72.59	7.77	320.53	0.61	6.42	2.78	0.30
	Boll stage	64.65	3.09	0.55	0.80	1.99	0.37	104.30
R^2^_adj_	Bud stage	-0.56	0.80	0.56	0.90	0.99	0.93	0.88
	Boll stage	0.72	0.71	-0.22	0.91	0.98	0.94	0.93

Note: logistic equation: y = A_2_ + (A_1_-A_2_)/(1 + (x/x_0_)^p).

#### Effect of hail fall density on boll falling rate

The results indicate that the boll falling rate increases with increased hail fall density at both the bud stage and the boll stage (S6 Fig), which is similar to the damage pattern for leaves and branches with hail fall density. When the average hail size was approximately 2.71 cm and the hail density was varied, the variation in the boll-falling rate was 0.36 to 0.65 during the bud stage and 0.22 to 0.37 during the boll stage. The minimum boll-falling rate occurred when the hail size was 1.0 cm: 0.20 during the bud stage and 0.09 during the boll stage. The maximum occurred when the hail size was 5.0 cm: 0.99 during the bud stage and 0.69 during the boll stage. Meanwhile, both the minimum and maximum boll-falling rates during the bud stage were greater than those during the boll stage. This indicates that regardless of hail size, the cotton bolls are more vulnerable to hail fall density during the bud stage than during the boll stage.

**Fig 6:**
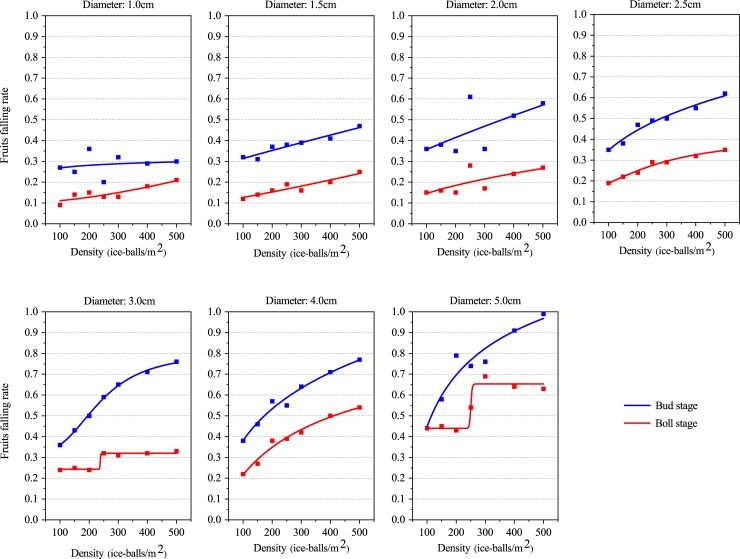
Logistic function between the boll falling rate and hail fall density.

A significant logistic function was found between the boll-falling rate and the hail fall density during both the bud stage and the boll stage, except in some cases (Fig 6, Table 7). However, the R^2^adj during the boll stage was less than 0.6 under most cases, and it was even negative when the hail size was 1.0 cm during the bud stage and 2.0 cm during the boll stage. This indicates that the logistic relation between the hail fall density and the boll-falling rate is relatively poor. However, the R^2^adj was more than 0.77 at both the bud stage and the boll stage when the hail size was 1.5 cm, 2.5 cm, 3.0 cm, 4.0 cm and 5.0 cm, and the R^2^adj exceeds 0.85 during the bud stage. Meanwhile, the RSS was less than 0.05 at both the bud stage and the boll stage, and it was always less than 0.01 during the boll stage (S1 Table). This indicates that the fit of the logistic function is superior at the bud stage when compared to the boll stage.

**Table 7:** Regression parameters of the logistic equations between the boll falling rate and hail fall density.

Regression parameter		1.0 cm	1.5 cm	2.0 cm	2.5 cm	3.0 cm	4.0 cm	5.0 cm
A_1_	Bud stage	-0.25	0.27	0.29	0.01	0.32	-0.40	-6.11
	Boll stage	0.10	0.16	0.07	0.15	0.24	-0.09	0.44
A_2_	Bud stage	0.54	8.51428E^6^	748.08	3.63	0.80	4.77	1.87
	Boll stage	655.52	6.17E^8^	2.81	0.41	0.32	0.95	0.65
X_0_	Bud stage	0.23	2.37E^6^	4.26E^6^	26081.87	235.12	27303.83	0.98
	Boll stage	141192.15	3.64E^6^	29009.80	262.46	238.21	297.06	250.29
P	Bud stage	0.10	0.94	0.87	0.41	2.90	0.31	0.33
	Boll stage	1.55	1.15	0.63	1.71	261.77	0.82	109.28
R^2^_adj_	Bud stage	-0.92	0.85	-0.13	0.93	0.99	0.95	0.86
	Boll stage	0.58	0.77	0.05	0.96	0.95	0.96	0.94

Note: logistic equation: y = A_2_ + (A_1_-A_2_)/(1 + (x/x_0_)^p).

## Discussion

### Experiment setting

This study evaluated the hazardous impacts of hail falls on cotton crops by simulating hailstorms using a new set of experimental equipment and procedures.

A specially designed hail hazard simulation apparatus was developed which is suitable for conducting field experiments in different regions. In previous studies, hailpad [33, 49, 50] and meteorological radar [48] were widely applied to obtain hail parameters or detect hailstorms at a relatively large scale. However, the apparatus consume huge manpower and financial resources, and the data produced by the apparatus typically must be combined with insurance loss data or field surveys to set up the relation between hail hazard intensity and loss. Therefore, hailpad and meteorological radar are only applicable in specific regions. However, our proposed apparatus is easily operated, can be set up in any region, and can be applied at any growth stage of plants. This enables a flexible layout and strong continuity in obtaining experimental data.

One of the main advantages of this apparatus is that diverse hail fall scenarios can be simulated. For example, the effects of strong winds on plants, which usually accompany hail, can be simulated from this experiment. Additionally, real ice balls, instead of stones, were used to simulate hailstorms. Furthermore, the landing speed and quantity of ice balls over an experimental plot can be precisely controlled. All these advantages enhance the verisimilitude of the simulated hail fall process. Hail fall simulations with more simple designs [25, 32], reapers [22], high-pressure water sprayers [23], scissors [24], and manually thrown ice balls or stones [27] have been widely applied in former studies to simulate hail strike. However, these devices are very difficult to control precisely, and they have little ability to simulate diverse hail fall scenarios. For example, the apparatus used by Seino (1985) [32] was simply composed of a container that holds stone balls above the land surface at a 3.4 m height, and it simulates hail fall by freely dropping a stone with diameter of 2.2 cm to the ground. In addition, these pioneer studies [22–25, 27, 32] seldom fully considered factors such as diameter, density (the hail number in unit area), and final landing speed in hail strike simulations. Although the results of these studies have improved our knowledge of hail-induced damages, they cannot accurately reveal the relation between hail hazard intensity and the damages caused by natural hail events.

Using the apparatus and experimental method designed in this paper, the hail hazard parameters and the hail-affected body damage data can be obtained at the same time. This facilitates the establishment of direct links between hail strike and crop damages. This link is significant for understanding the mechanisms leading to damages by hail strike [36]. However, our proposed technique has its limitations. First, the hailstorm scenarios are limited. For example, the diameter of hail stones is concentrated within 1–5 cm, and the hailfall density is concentrated within 100–500 pieces/m^2^. In addition, each hail fall scenario can only utilize hail stones with a single diameter, while real hailstorms include a combination of various hail diameters. Furthermore, the spatial coverage of our study is limited to the field scale. The reliability of the method for estimating hail damage over a larger landscape requires further investigated. Therefore, the hailstorm scenarios should be further improved to more accurately simulate real hailstorms and improve the quality of crop damage assessments. To accomplish this aim, additional experiments at larger scales and in different regions are also necessary. Although technologies such as aerial multispectral imaging [51] and radar [52] have shown great potential for detecting hail and corresponding crop damage, these methods still require a significant amount of field data to verify and improve the detection accuracy. By combining data from aerial multispectral imaging, radar, and field experiments, hail damage over large landscapes could be detected with greater rapidity and precision.

### Cotton damages and hail fall parameters

Our results show that the defoliation rate, branch injury rate, and boll falling rate increase as the hail diameter increases, regardless of the hail fall density. These results agree with previous reports that suggest that larger hail diameters result in higher levels of damages or losses [33, 49, 50, 53, 54]. For example, Garcia et al. (1976) [50] pointed out that hail ball size is a good indicator for estimating hail-induced losses. Shan (1998) [54] also found that larger hail ball sizes are associated with more serious the disasters. Therefore, there is a strong positive correlation between hail size and cotton damages.

In contrast, hail fall density has less of an impact on cotton damage than hail size, indicating that hail diameter is a more suitable factor for predicting cotton damages. However, Garcia et al. (1976) [50] demonstrated that hail fall density and crop damages exhibit a strong relationship. Our results also indicate that the cotton defoliation rate, the branch falling rate, and the boll falling rate increased with the increasing hail density. However, the logistic functions for various types of cotton damage and hail fall density exhibited lower accuracy than the relationships between hail diameter and cotton damages. This suggests that the hail fall density could be used a cotton vulnerability assessment indicator, but it should not be used as the priority indicator. Some studies indicate that hail diameter is the main indicator for deriving crop losses [53–55]. Our results also suggest that it can be used as a priority indicator for predicting cotton damages/losses caused by hail hazard.

There have been numerous studies on how crop damages impact crop yield, such as cotton [25–29], potato [23, 30, 31], onion [21], soybean [24, 32], wheat [32, 33], lentil [34], and guar [22]. Few of these studies focused on the relationship between hail fall characteristics and crop damage, i.e., scientific knowledge of plant physiology related to damage by hail. Some studies investigated the relation of crop damage with hail size [53, 55] and hail kinetic energy [48]. However, the damage in these papers still focuses on yield loss rather than crop characteristics, and hailstorm kinetic energy was used rather than hail diameter and hail fall density. These studies indicate a need for additional efforts to quantify the relationships of hailstorm parameters with crop damage. This research effort is needed in many other plants besides cotton. These relations are incredibly significant for quickly predicting potential losses after hailstorms, which can be used for agricultural insurance and re-insurance to mitigate hail deduced disaster risk [6, 8, 22, 26, 27].

### Cotton damages/losses and growth stages

Previous studies indicated that crops exhibit different levels of resilience to hail hazard at different growth stages, which leads to differential damage degrees and corresponding crop losses. For example, Wille and Kleinkopf (1992) [30] simulated the effect of hail on potatoes, and they found that the yield and quality were mainly affected by the damage during the vegetative growth stage. Furthermore, Irigoyen et al. (2011) [23] pointed out that damage produced during tuber formation or flowering considerably reduced yield, while defoliations that occurred after tubers had completed growth barely affected yields. Sij et al. (2005) [22] found higher reductions in guar yield when hail events occurred during the early flowering stage as compared to those that occurred during the maturation stage. Lang et al. (2007) [56] found that hail events that occurred during the tasseling stage induced the most serious yield reductions in corn. For lentil, yield decreased as damage intensity increased, and most yield reduction was seen when the damage occurred in reproductive growth [34]. Conley et al. (2009) [24] found that node removal in the soybean development stage must be considered when estimating soybean seed yield loss; soybean oil content was only affected by extreme node removal treatments while protein content was unaffected. From above, we can conclude that crop damages occur within the later vegetative growth stage and the early reproductive growth stage, for example, tuber formation or flowering for potatoes, the early flowering stage for guar, and the tasseling stage for corn most seriously reduce the crop yield/quality. Of course, the effects of hail damage in different growth stages on crop yield and quality need to be analyzed on a crop-by-crop basis.

It is clear that the impacts of hail damages on crop yield are complicated. Cotton has proved to have a strong ability to recover from disturbances, such as hailstorms and pests. Lane (1959) [25] found that early-season stem injuries did not affect cotton growth and fruiting. Wilson et al. (2003) [57] reported that crop yield was almost unaffected by damages, such as defoliation, when they were applied before first flower buds appeared or early fruit loss, even under treatments with very heavy damage. It was also found that the quality characteristics of the fiber were negligibly affected [26]. This is because the plants overcame the leaf area disparity via an accelerated ontogeny of main stem leaves [29]. However, the degree of recoverability decreased as the severity of damage increased or as the same damage was applied to older plants [26]. Cotton can still obtain considerable yield if hail hazard occurs more than 40 days prior to the budding period [28]. Therefore, these studies demonstrated that hail events that occur at the early stage or after maturity may not result in significant cotton yield reductions.

This paper does not discuss the effects of hail-induced crop damages on cotton yield and quality. However, our results demonstrate that cotton at the budding stage is more vulnerable than at the boll stage. Given the same intensity of hail strike, cotton in the bud stage will receive a higher level of damage. By integrating the findings of previous research and this study, we argue that crop damages caused by hail events in the bud stage may lead to higher yield reductions. Li and Zhao (2006) [28] concluded that hail events that occurred in the bud stage caused less cotton yield reductions than hail events that occurred in other stages. They further pointed out that the effect of hail on the cotton yield decreases as the period between the hail event and the bud stage increases. Therefore, we conclude that the bud stage is the key stage for hail defense in cotton.

In summary, our results clearly indicate that cotton vulnerability to hail hazard changes between growth stages. Prior studies mainly focused on how crop yield changes with various growth stages and hail-induced damages, while this paper focus on how damages to cotton vary with various hail sizes and hail fall densities. The relationship between hail fall parameters and crop damages revealed in this paper will benefit the comprehensive understanding of hail impacts on cotton damages, yield, and quality.

### Future work and shortcomings

Since different crops have different physiological properties, determining the mechanism by which crop damage impacts yield and quality is very complicated. For example, studies show that early season cotton damage produced almost no residual effect on cotton lint yield [25, 26, 28, 57]. A 51% loss in leaf area only resulted in a 9% decrease in yield [29]. This might be explained by affected plants overcoming the leaf area disparity via an accelerated ontogeny of main stem leaves [29]. However, the degree of recoverability is influenced by multiple factors, e.g., hail hazard intensity, growing season length, the type of damage, and field management. The length of the growing season also plays a very important role in determining the degree to which hail damage will impact crop yield. For instance, Wilson et al. (2003) [57] reported that a high level of damage can result in no impact on yield if the season length allows regrowth. However, they also reported that a low level of damage late in the season may lead to significant yield reduction for the crop. Therefore, a comprehensive analysis is needed to reveal the complicated impact of crop damage on cotton yields.

Due to length limitations, this study is focused on revealing the relation between hailstorm parameters and cotton damage, instead of assessing the effects of hail-induced damages on cotton yield and quality. As part of a separate research project, we also systematically collected the yield data for the cotton subjected to the various hail fall scenarios. Therefore, our study provides solid support for the comprehensive analysis of the quantitative relationships between hail storm-induced cotton damages and cotton yields. There is still room for improving this study by applying novel analytical methods. For example, we did not completely separate the respective effects of hail diameter and hail density on cotton damages. To address this issue, analytical methods, such as natural log transformation of the observed data, stepwise regression to build a more comprehensive predictive model, and the Copula model [58], may provide insights. In summary, further analysis of the impacts of hail-induced crop damages on yield and fiber quality via these analytical methods will deepen our understanding of the vulnerability of cotton subjected to hail hazard. We hope to conduct these analyses in subsequent studies.

## Conclusions

Determining cotton vulnerability to hail hazard is a key scientific issue for hail disaster mitigation in China and across the world. This paper aims to reveal the effects of hail parameters on crop damages via field experiments. The following conclusions can be drawn.

We develop a set of apparatus and the corresponding experimental method to conduct the field study of cotton hail hazard. This apparatus is capable of shooting 0.5–10.0 cm ice balls at a speed ranging from 20 m/s to 45.0m/s. The shooting direction can range from 0° to 120° vertically and 0° to 180° horizontally. Using this apparatus, we designed detailed hail fall scenarios based on the variables of cotton growth stage, hail size, and hail fall density. Our results show that the novel apparatus and method for simulating hail damage increase experimental precision compared to previous studies. Moreover, the apparatus and experimental method presented in this paper can reproduce hailstorms with a higher level of verisimilitude than previous methods, and it can collect a variety of data. This novel approach enables the possibility to comprehensively analyze the effects of hail parameters and hail-induced crop damages on yield and quality.

The cotton damages caused by hail exhibit different relationships with hail diameter and hail fall density and the cotton damages also vary with different growth stages. The results show that the defoliation rate, branch injury rate, and boll falling rate increase with increasing hail diameter and hail fall density. This means that greater damage to cotton plants is associated with larger hail diameter and hail fall density. Our results also indicate that cotton at the bud stage is more sensitive to hail strikes, which implies that the bud stage is the key growth stage to prevent and reduce cotton damages from hail hazard. Hail diameter and hail fall density both have significant logistic relationships with cotton damages at both the bud and boll stages. However, they differ greatly in the ability to predict cotton damages. Based on the results of present research, we argue that hail diameter can be used as the priority indicator to predict cotton damages from hail falls.

The quantitative relationships between hail fall parameters and cotton damages revealed in this paper are sound supplements to previous studies and aid understanding of cotton vulnerability to hail hazard. Nevertheless, further study is needed to comprehensively understand the effects of hail-induced crop damages on cotton yield.

## Supporting information

S1 Fig Variation in the defoliation rate with variation in hail size. (EPS)

S2 Fig Variation in branch breaking rate according to hail size. (EPS)

S3 Fig Variation in the boll falling rate according to hail size. (EPS)

S4 Fig Variation in the defoliation rate according to hail fall density. (EPS)

S5 Fig Variation in the branch injury rate according to hail fall density. (EPS)

S6 Fig Variation in the boll falling rate according to hail fall density. (EPS)

S1 Table Residual error sum of squares (RSS) of the predicted crop damages (using the fitted logistic functions) compared with the observed crop damages. (DOCX)
