# Supplementary material for: Vulnerability of cotton subjected to hail damage
Source: PLoS One. 2019 Jan 30;14(1):e0210787. doi: 10.1371/journal.pone.0210787 (PMC6353171; doi:10.1371/journal.pone.0210787)
Supplement: S1 Table — (DOCX) [file pone.0210787.s007.docx]

**Table 1.** Residual error sum of squares (RSS) of the predicted crop damages (using the fitted logistic functions) compared with the observed crop damages.

| Hail diameter-defoliation rate | | | | | | | |
| --- | --- | --- | --- | --- | --- | --- | --- |
|  | 100 particles/m^2^ | 150 particles/m^2^ | 200 particles/m^2^ | 300 particles/m^2^ | 400 particles/m^2^ | 450 particles/m^2^ | 500 particles/m^2^ |
| Bud stage | 0.00284 | 0.01146 | 0.02841 | 0.01251 | 0.00799 | 0.01474 | 0.00186 |
| Boll stage | 0.00797 | 0.0036 | 0.00205 | 0.0053 | 0.00381 | 0.00113 | 0.00711 |
| Hail diameter-branch breaking rate | | | | | | | |
| Bud stage | 0.00395 | 0.00418 | 0.00975 | 0.04153 | 0.00265 | 0.0055 | 0.0013 |
| Boll stage | 0.00432 | 5.776E^-4^ | 0.00149 | 0.00543 | 0.00399 | 0.00164 | 0.00251 |
| Hail diameter-fruits falling rate | | | | | | | |
| Bud stage | 0.00164 | 0.0028 | 0.0054 | 0.03056 | 0.00945 | 0.00981 | 0.0107 |
| Boll stage | 0.00821 | 0.00359 | 0.00209 | 0.00381 | 0.00492 | 0.00188 | 0.00431 |
| Hail fall density-defoliation rate | | | | | | | |
|  | 1.0 cm | 1.5 cm | 2.0 cm | 2.5 cm | 3.0 cm | 4.0 cm | 5.0 cm |
| Bud stage | 2.36901E^-4^ | 0.00115 | 0.02377 | 0.00187 | 0.00287 | 0.00321 | 0.0588 |
| Boll stage | 0.00346 | 2.01716E^-4^ | 0.00715 | 2.2486E^-4^ | 0.00185 | 0.00141 | 3.33333E^-4^ |
| Hail fall density-branch breaking rate | | | | | | | |
| Bud stage | 0.01852 | 0.00126 | 0.01597 | 0.00309 | 6.81898E^-4^ | 0.00682 | 0.0111 |
| Boll stage | 5.2E^-4^ | 9.10846E^-4^ | 0.00683 | 5.47478E^-4^ | 9.29685E^-5^ | 0.00154 | 0.00267 |
| Hail fall density-fruits falling rate | | | | | | | |
| Bud stage | 0.01516 | 0.00129 | 0.04435 | 0.00169 | 2.3782E^-4^ | 0.00283 | 0.01456 |
| Boll stage | 0.00188 | 0.00127 | 0.00963 | 4.06121E^-4^ | 2.66667E^-4^ | 0.00168 | 0.00227 |
